# IGF-1R, a target of let-7b, mediates crosstalk between IRS-2/Akt and MAPK pathways to promote proliferation of oral squamous cell carcinoma

**DOI:** 10.18632/oncotarget.1812

**Published:** 2014-03-21

**Authors:** Ling Gao, Xiaolong Wang, Xiaofei Wang, Linmei Zhang, Cui Qiang, Su'e Chang, Wenhao Ren, Shaoming Li, Yang Yang, Dongdong Tong, Cheng Chen, Zongfang Li, Tusheng Song, Keqian Zhi, Chen Huang

**Affiliations:** ^1^ Department of Oral Maxillofacial Surgery, Stomatology Hospital of Xi'an Jiaotong University College of Medicine, Xi'an, Shaanxi, P. R. China; ^2^ Key Laboratory of Environment and Genes Related to Diseases, College of Medicine, Xi'an Jiaotong University, Xi'an, Shaanxi, P. R. China; ^3^ Department of General Surgery, the Second Affiliated Hospital, School of Medicine, Xi'an Jiaotong University, Xi'an, Shaanxi, P. R. China

**Keywords:** oral squamous cell carcinoma, IGF-1R, IRS-2, let-7b, proliferation

## Abstract

Insulin-like growth factor (IGF) signaling is involved in oral squamous cell carcinoma (OSCC), but IGF-1 receptor (IGF-1R)-mediated intricate regulatory networks among molecular interactions and signalling path ways in OSCC remain unclear. Here, we found that overexpression of IGF-1R and insulin receptor substrate-2 (IRS-2) was negatively associated with histological differentiation. IGF signaling stimulated OSCC cell growth. Conversely, overexpression of let-7b inhibited proliferation and colony formation and triggered S/G2 cell cycle arrest by targeting IGF-1R and IRS-2 through the Akt pathway. Also, the inverse relationship between expression of let-7b and IGF-1R/IRS-2 was confirmed in OSCC tumor xenografts and clinical specimens. Furthermore, by activating ERK1/2, IGF-1R transcriptionally upregulated IRS-2. Our results indicate that let-7b/IGF-1R-mediated crosstalk between IRS-2/Akt and MAPK is involved in OSCC and is a potential therapeutic target for therapy.

## INTRODUCTION

Oral cancer is the eighth most common malignancies in the head and neck with over 145,500 deaths annually worldwide [International Agency for Research on Cancer (IARC; 2011)][1] and oral squamous cell carcinomas (OSCC) accounts approximately for 90% of all oral cancers. Despite of the numerous advances in cancer treatment, the 5-year survival rate of OSCC patients is 50%, which remains unchanged over the last decade[2]. Recently, several environmental factors, including tobacco smoking, betel nut chewing and human papillomavirus (HPV) infection, are the main causes of OSCC[3, 4]. However, not everyone exposed to these predisposing factors develops oral cancer. Many studies have emerged that oral carcinogenesis arises as a result of the oncogenes activation or tumor suppressor genes inactivation[5]. Therefore, a better understanding of regulatory networks among molecular interactions and signalling pathways is essential for identification of novel prognostic markers or therapeutic targets for OSCC.

Insulin-like growth factor 1 receptor(IGF-1R) is a transmembrane receptor tyrosine kinase mainly activated by IGF1 or IGF2 through autocrine and paracrine, which has kinase-independent biologic functions[6,7]. The activated IGF-1R binds to adaptor molecules such as insulin receptor substrates(IRSs) and Shc and then triggers multiple downstream signaling cascades, including phosphatidylinositol 3-kinase (PI3K)/Akt and mitogen-activated protein kinase(MAPK) signaling pathways, which regulate oncogenic transformation, growth and survival of cancer cells[8,9]. Furthermore, IRS-2 can integrate signals from multiple kinases other than IGF-1R, such as PI3K and Src[10], which subsequently phosphorylates Grb2-associated binding partner(GAB), the resultant activation of c-Src and Ras leads to up-regulation of MAPK signal pathway[11]. ERK1/2 can subsequently phosphorylate GAB1 on several serine residues that are adjacent to p85 PI3K-binding sites and then develop GAB-p85 PI3K complexes to influence the Akt pathway[12]. However, the role of crosstalk between IRS-2/Akt and MAPK signal pathways via IGF-1R at transcriptional level in modulating tumorigenesis remains largely unknown. Recent studies have demonstrated that aberrant expression of IGF-1R was correlated with tumor growth and poor prognosis in several human cancers such as myeloma[13] and breast cancer[14]. On the other hand, IGF-1R, as a hallmark of cell carcinogenesis, could also be regulated by transcription factors(TFs)[15], methylation[16] and microRNAs(miRNAs)[6, 17].

miRNAs are an abundant class of short(21–23 nt), non-coding RNAs that post-transcriptionally mediate the regulation of target genes via sequence-specific interactions with 3'-untranslated regions (3'-UTR) of several target mRNAs to induce their degradation or translational repression. Recently, more than 50% of the miRNAs are located in cancer-associated genomic regions or fragile sites[18] and are identified as classical oncogenes or tumor suppressor genes[19, 20], suggesting the pivotal roles of miRNAs in the pathogenesis of human cancers. In OSCC, aberrant expression of miRNAs, such as miR-24[21] and miR-218[22], have been demonstrated to regulate tumor cell proliferation, apoptosis and invasion by targeting downstream proteins. let-7b, a member of the let-7 family, has been reported to be downregulated in human cancers, including oral cancer[23], as it suppresses the expression of several oncogenes such as c-Myc[24] and ER-α[25]. Nevertheless, whether let-7b is mechanistically associated with OSCC progression remains unclear.

Based on a bioinformatics analysis, IGF-1R and IRS-2 are predicted to be mutual targets of let-7b. In this study, we identified significant downregulation of let-7b and simultaneous upregulation of IGR1R, which triggered the PI3K pathway that in turn led to accelerated OSCC cells proliferation both *in vitro* and *in vivo*. More importantly, we also found that IGF-1R was not only able to induce direct phosphorylation of IRS-2 protein, but also regulated intranuclear transcription of IRS-2 through the MAPK pathway.

## RESULTS

### Overexpression of IGF-1R and IRS-2 were inversely associated with poor histology of OSCC

**Figure 1:**
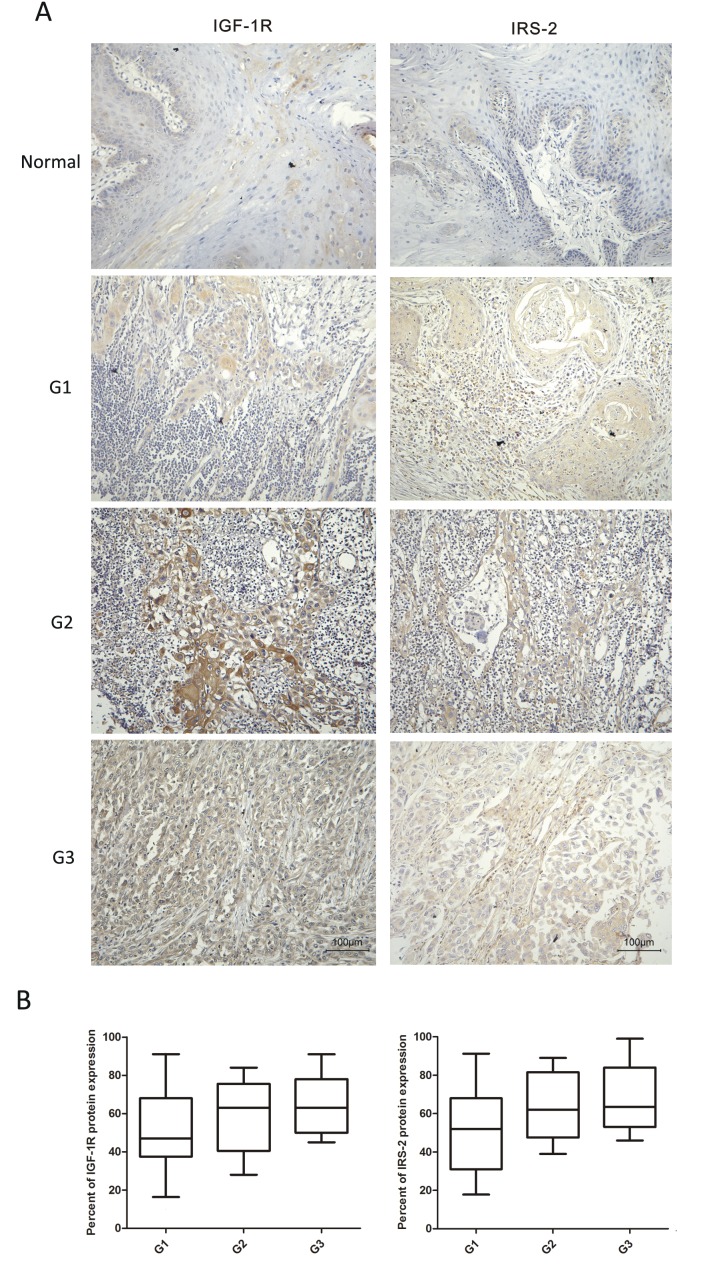
IGF-1R and IRS-2 were overexpressed and inversely correlated with tumor histology in OSCC samples A, Protien levels of IGF-1R and IRS-2 in different histological types of OSCC specimens and normal tissues were measured by IHC staining(20×). B, The percent of IGF-1R(left) and IRS-2(right) protein expression in different histological grade of OSCC specimens. Tumor histological grade depended on the AJCC criteria: grade 1 (G1), well differentiated; grade 2 (G2), moderately differentiated; and grade 3 (G3), poorly differentiated. For B, Whiskers represented the 5^th^ and 95^th^ percentiles.

To evaluate the potential clinical significance of IGF-1R and IRS-2 expression, we examined the levels of IGF-1R and IRS-2 expression by IHC staining in 64 OSCC samples and 20 normal tissues. As shown in Figure 1, in contrast to the decreased expression of let-7b in OSCC samples as compared to the normal tissues, the expression levels of IGF-1R and IRS-2 in OSCC smaples were significantly increased. Correlation between IGF-1R and IRS-2 expression levels and clinicopathological characters of OSCC patients was summarized in Table 1. Strikingly, the high IGF-1R (well:40.5% (15/37); Moderate:61.5% (8/13); poor:85.7% (12/14)) and IRS-2(well:56.8%(21/37); Moderate:69.2%(9/13); poor:92.9%(13/14)) levels were significantly associated with poor tumor histology(*P*=0.013, *P*=0.036, respectively), but not with age, gender and TNM stage, suggesting that upregulated IGF-1R and IRS-2 proetin expression might be involved in the progression of OSCC.

**Table 1:** Patient characteristics and clinicopathologic correlation of IGF-1R and IRS-2 expression

Characteristics	Number of cases	IGF-1R expression		P-value	IRS-2 expression		P-value
		High	Low		High	Low	
Age (years)				0.304			0.659
≥60	31	19	12		20	11	
<60	33	16	17		23	10	
Gender				0.418			0.614
Male	43	22	21		28	15	
Female	21	13	8		15	6	
Histology				0.013			0.036
Well	37	15	22		21	16	
Moderate	13	8	5		9	4	
poor	14	12	2		13	1	
pTNM Stage				0.270			0.554
I	15	5	10		8	7	
II	18	11	7		13	5	
III	11	6	5		7	4	
IV	20	13	7		15	5	

### let-7b was downregulated in OSCC

**Figure 2:**
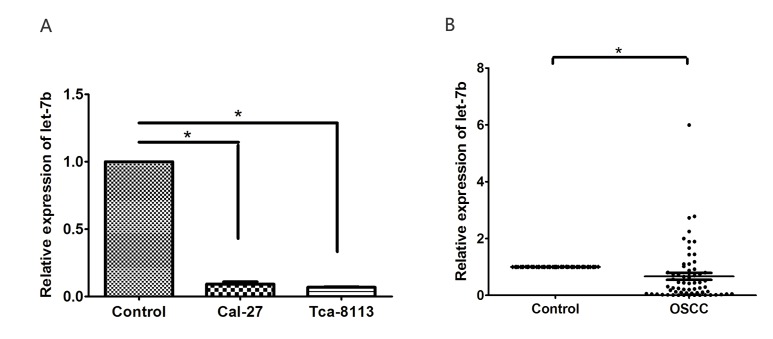
The expression of let-7b was reduced in OSCC cell lines and clinical sepecimens A, qRT-PCR analysis of let-7b expression in normal oral mucosa cells and OSCC cell lines (Cal-27, Tca-8113). B, The average expression level of let-7b in OSCC (n=64) and normal tissues (n=20). The expression of let-7b was normalized to U6 RNA. Data were from three independent experiments and represented as mean ± SEM. *P<0.05.

IGF-1R and IRS-2 are predicted as two of the candidate targets of let-7b using bioinformatics analysis (TargetScan, PicTar and DIANA-microT-CDS). To evaluate the role of let-7b in the development and progression of OSCC, let-7b expression was examined in OSCC cell lines and clinical specimens. qRT-PCR showed that let-7b expression was significantly decreased in OSCC cell lines(Cal-27 and Tca-8113) compared with normal oral mucosa cells (*P*<0.001) (Figure 2A).

We further investigated the expression level of let-7b in 64 OSCC specimens and 20 normal tissues by qRT-PCR. Most OSCC patients(48 of 64, 75%) showed lower let-7b levels with respect to normal counterparts(0.0005- to 0.921-fold change). Consistent with the results examined from OSCC cell lines, the average expression of let-7b was remarkably lower in OSCC specimens than normal tissues(*P*=0.008, Figure 2B). In addition, as shown in Supplementary Table S3, even though no significant correlation was found between let-7b expression and clinicopathologic characteristics of OSCC patients, we supposed that let-7b may act as a potential tumor suppressor in OSCC.

### let-7b directly targeted the IGF-1R and IRS-2

**Figure 3:**
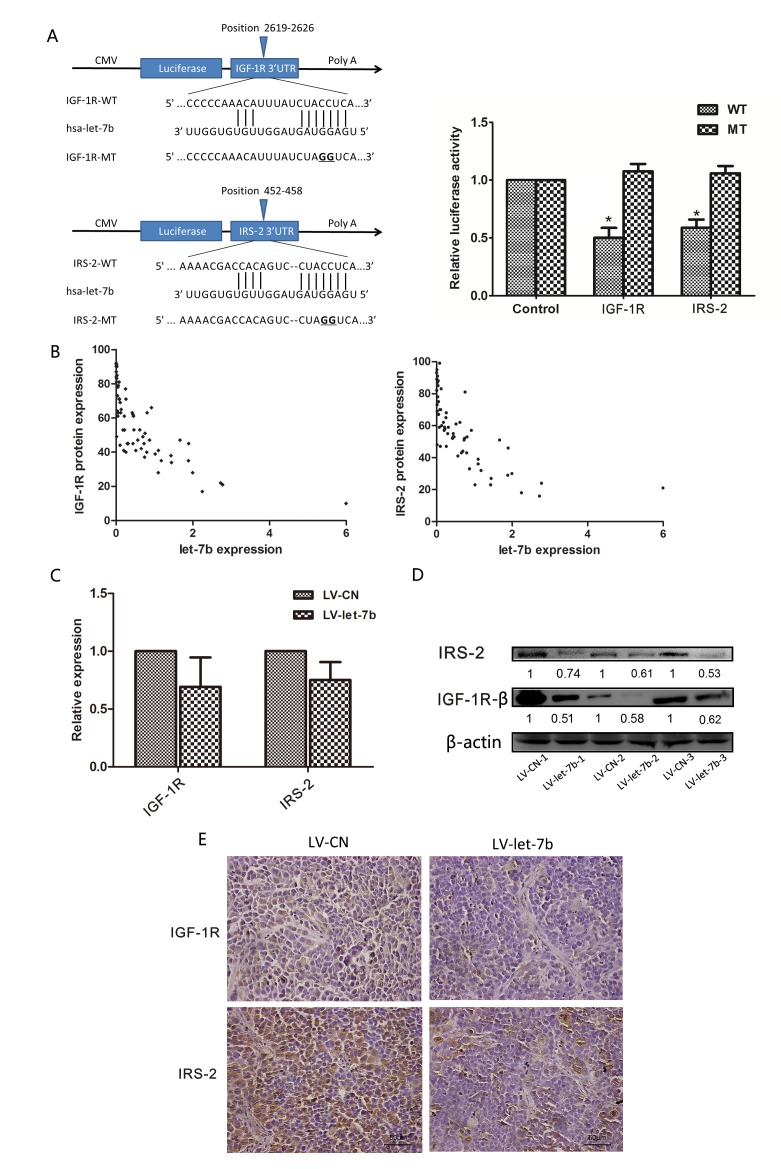
let-7b downregulated protein expression of IGF-1R and IRS-2 by targeting their 3'UTRs A, Putative let-7b-binding sites in the IGR1R (left-upper) and IRS-2 (left-lower) 3'UTRs, mutations were generated in the IGF-1R and IRS-2 3'UTR sequences by mutating 2nt for the seed region of let-7b, as indicated. The wild-type or mutant 3'UTRs(indicated as WT or MT) were cloned into the downstream of the luciferase reporter vector and then cotransfected into HEK293 cells with pre-let-7b. The relative luciferase activities were measured 24h posttransfection and normalized by calculating the ratio of firefly luciferase with *Renilla* luciferase activity (right). B, The linear regression between let-7b and IGF-1R (left), IRS-2 (right) protein expression levels was analysed. IGF-1R, IRS-2 protein and let-7b expression levels were evaluated by immunohistochemistry staining and qRT–PCR for 64 OSCC specimens, respectively. C~E, LV-let-7b- and LV-CN- infected tumors were excised from mice, the expression levels of IGF-1R and IRS-2 mRNA were measured by qRT–PCR (upper-left) and the protein expression levels of IGF-1R and IRS-2 were analyzed by Western blot (upper-right) and immunohistochemistry staining (40×)(lower), respectively. The ratios of detected target proteins against β-actin were shown under each Western blot image. Data represented the mean ± SEM of three independent experiments. *P<0.05.

To experimentally confirm whether let-7b directly targets IGF-1R and IRS-2 in OSCC cells, we constructed 3'-untranslated region (UTR) fragments of IGF-1R and IRS-2, a binding site of let-7b, in which wild-type(IGF-1R-WT, IRS-2-WT) and mutant(IGF-1R-MT, IRS-2-MT) binding sites were subcloned into the region downstream of the pmiRGLO dual-luciferase reporter vector (Figure 3A). HEK293 cells were then cotransfected with pre-let-7b and WT- or MT-3'UTR vector, a remarkable reduction of luciferase activities of WT-3'UTRs(IGF-1R and IRS-2) reporter constructs was observed in HEK293 cells with pre-let-7b transfection (*P*=0.038, *P*=0.039, respectively), but let-7b failed to inhibit the luciferase activity of the reporter vector containing mutant binding sites, indicating that let-7b could bind directly to the 3'-UTR of IGF-1R and IRS-2.

Furthermore, we examined the correlation of let-7b expression level from qRT–PCR analysis with the protein levels of IGF-1R and IRS-2 from immunohistochemistry analysis on FFPE specimens of 64 OSCC tissues. As Figure 3B showed, the degree of let-7b downregulation inversely correlated with the extent of upregulated expression of IGF-1R and IRS-2 by spearman's correlation analysis (r=-0.717,*P*<0.001, r=-0.696, *P*<0.001, respectively), suggesting that the target effect of let-7b on the IGF-1R and IRS-2 was clinical relevant in OSCC samples.

We also evaluated the association between let-7b and IGF-1R, IRS-2 in harvested tumors excised from mice. qRT-PCR showed let-7b overexpression had no effect on IGF-1R and IRS-2 mRNA levels(Figure 3C), however, the protein levels of IGF-1R and IRS-2 were significantly reduced in tumors injected with LV-let-7b compared with control group by Western blot and immunohistochemistry (Figure 3D and E). Taken together, these results proved that let-7b post-transcriptionally regulated protein expression levels of IGF-1R and IRS-2 by directly targeting their 3'UTRs.

### let-7b induced growth inhibition of Tca-8113 cells ***in vitro*** and ***in vivo*** via IGF-1R and IRS-2

**Figure 4:**
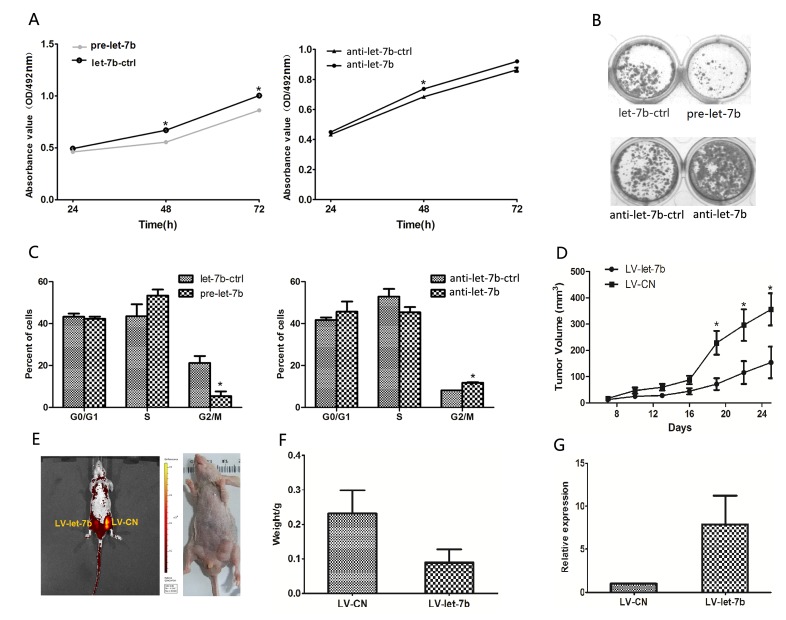
IGF-1R and IRS-2-mediated let-7b inhibited OSCC cell growth *in vitro* and *in vivo* A, At 24, 48 and 72h after transfection with pre-let-7b, let-7b-ctrl, anti-let-7b or anti-let-7b ctrl, cell proliferation was examined by the MTT assay. All data were shown as mean ± SEM. *P<0.05, **P<0.01. B, Representative micrographs of crystal violet-stained cell colonies were analyzed by colony formation assay at day 15 after transfection. C, The histograms for cell-cycle distribution of Tca-8113 cells transfected with miRNAs for 24h based on the flow-cytometric analysis, data were presented as mean ± SEM. *P<0.05. D, Tca-8113 cells were infected with LV-let-7b and LV-CN injected s.c. into nude mice, growth curve of tumor volume was formed every 3 days for 18 days(n=10). Each data point represented mean ± SEM. E, At day 25, tumor growth was measured by *in vivo* bioluminescence imaging. F, The mice were anesthetize and sacrificed at the experimental endpoint and tumors infected with LV-let-7b and LV-CN were weighted at day 25 after the initial injection. Data were represented as mean ± SEM. G, Levels of let-7b expression in tumor xenografts were quantified by qRT-PCR. Each data point represented mean ± SEM. *P<0.05.

In the attempt to confirm whether the effects of IGF-1R and IRS-2-mediated let-7b on proliferation observed in OSCC cells and clinical specimens were related to tumor growth. First, Tca-8113 cells were transfected with pre-let-7b or anti-let-7b, respectively. As shown in Figure 4A, the MTT assay displayed that cell growth was inhibited in pre-let-7b-transfected Tca-8113 cell, but promoted by transfecting Tca-8113 cells with anti-let-7b, the let-7b control or anti-let-7b control had no effect on cell growth. Similarly, the colony formation was suppressed in Tca-8113 cells transfected with pre-let-7b (Figure 4B).

In addition, the cell-cycle was simultaneously performed by flow cytometry and let-7b overexpression was found to trigger an accumulation of cells at S phase and a decrease in the percentage of cells in G2 phase (*P*=0.017), the phenomenon was opposite when Tca-8113 cells were transfected with anti-let-7b (*P*=0.05) (Figure 4C). These results suggested that let-7b blocked the S/G2 transition in Tca-8113 cells *in vitro*. To determine whether the growth-suppressive effect of let-7b was mediated by repression of IGF-1R and IRS-2 in OSCC cells, we transfected Tca-8113 cells with pre-let-7b, in combination with IGF-1R or IRS-2 plasmid. As shown in Supplementary Figure S1, let-7b overexpression induced cell growth inhibition and cell-cycle arrest, which was reverted by cotransfection of IGF-1R or IRS-2 plasmid.

To further verify the impact of let-7b on OSCC growth *in vivo*, we used lentiviral vectors to stably restore the expression of let-7b in Tca-8113 cells. The LV-let-7b-infected and LV-CN-infected cells were injected s.c. into both posterior flanks of nude mice. Palpable tumors were developed at day 7 and measured every 3 days. As shown in Figure 4D, tumor growth was significantly suppressed by LV-let-7b compared with the LV-CN during the experiment. In parallel, this trend was also confirmed by the sizes and weights tumors excised from animals. At day 25, the average volume of LV-Ctrl-infected tumors was ~2.31-fold larger than LV-let-7b tumors and average weight in LV-CN group was 0.23mg compared with the average weight of 0.09mg in LV-let-7b group(Figure 4E and F). Furthermore, we assessed the let-7b expression in tumor xenografts using qRT-PCR and found that tumors injected with LV-let-7b had ~2.21-fold increase of let-7b than the LV-Ctrl group (Figure 4G). Based on these findings, together with Figure 3, we concluded that let-7b could regulate OSCC cell proliferation by directly targeting the IGF-1R and IRS-2.

### Downregulation of IGF-1R and IRS-2 inhibited OSCC cell growth

**Figure 5:**
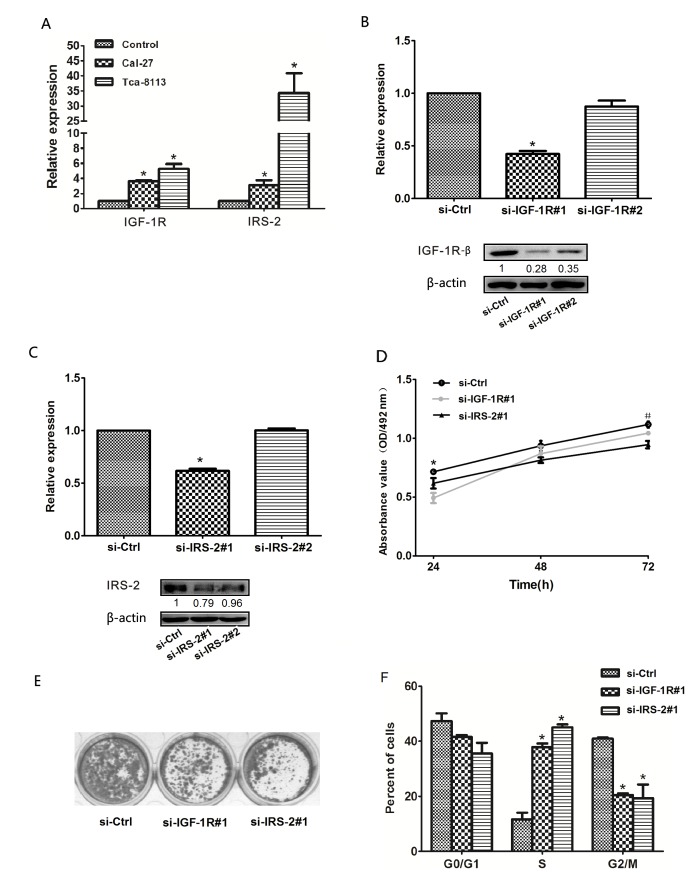
Reduced expression of IGF-1R or IRS-2 suppressed OSCC cell growth A, The expression levels of IGF-1R and IRS-2 in OSCC cell lines(Cal-27, Tca-8113) compared to normal oral mucosa cells using qRT–PCR. B, The expression level of IGF-1R was measured by qRT–PCR (upper) and Western blot (lower) in Tca-8113 cells transfected with 2 sets of specific si-IGF-1R, * *P*<0.05. C, the mRNA (upper, qRT–PCR) and protein(lower, Western blot) expression levels of IRS-2 in Tca-8113 cells transfected with 2 sets of specific si-IRS-2, * *P*<0.05. D, MTT assays was performed on days 24-72h after the transfection of Tca-8113 cells with si-IGF-1R, si-IRS-2 and negtive control(si-Ctrl), *si-IGF-1R, *P*<0.05; ^#^si-IRS-2, *P*<0.05, compared with si-Ctrl. E, The colony formation assay was performed at day 15 after transfection of Tca-8113 cells with si-IGF-1R, si-IRS-2 and negtive control(si-Ctrl). F, The representative histograms for cell-cycle distribution of Tca-8113 cells transfected with si-IGF-1R, si-IRS-2 and negtive control(si-Ctrl) for 24h. All numerical data represented mean ± SEM of 3 independent experiments.

To explore whether downregulation of IGF-1R and IRS-2 repressed the progression of OSCC cells. First, we identified that the IGF-1R and IRS-2 were significantly increased in OSCC cell lines(Cal-27 and Tca-8113) compared to norm al oral mucosa cells using qRT–PCR(Figure 5A), which were consistent with their protein expression levels measured by IHC staining. We next separately used 2 sets of specific small interfering RNAs (si-RNAs) against IGF-1R(si-IGF-1R) and IRS-2(si-IRS-2) to reduce expression levels of IGF-1R and IRS-2 in Tca-8113 cells, as assessed by qRT–PCR and Western blot. As shown in Figure 5B and C, si-IGF-1R#1 and si-IRS-2#1 significantly reduced the mRNA and protein levels of IGF-1R and IRS-2 and were used in subsequent experiments. Consistent with patterns of overexpression of let-7b, silencing of IGF-1R or IRS-2 inhibited cellular viability (Figure 5D) and colony formation (Figure 5E) and increased significantly the percentages of cells in S-phase but decreased proportions of G2-phase cells compared with control siRNA-transfected cells(Figure 5F). These data suggested that IGF-1R and IRS-2 promoted the S-G2 transition of cell-cycle progression and consequently aroused the proliferation of OSCC cells.

### IGF-1R enhanced the expression of IRS-2 via the promotion of ERK1/2 phosphorylation

**Figure 6:**
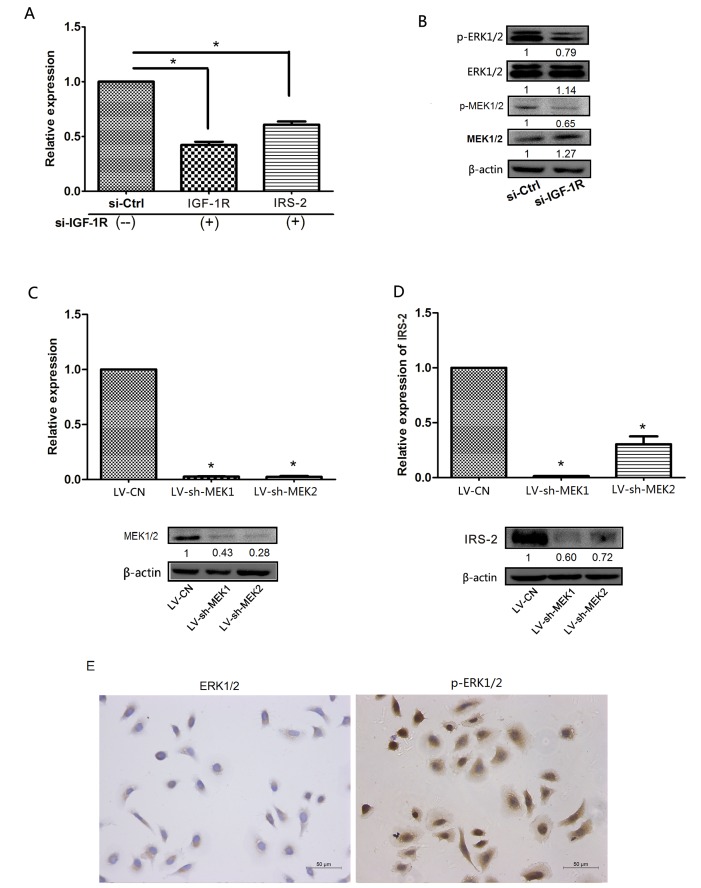
IGF-1R increased the expression of IRS-2 through the promotion of ERK1/2 phosphorylation A, The mRNA expression levels of IGF-1R and IRS-2 were measured by qRT–PCR in Tca-8113 cells transfected with si-IGF-1R and si-Ctrl at 24h. B, The protein expression levels of MAP2K1/2, p-MEK1/2, ERK1/2 and p-ERK1/2 were measured by Western blot in Tca-8113 cells transfected with si-IGF-1R and si-Ctrl at 24h. C, The expression levels of MEK1 and MEK2 were measured by qRT–PCR (upper) and the protein expression levels of MEK1/2 were assessed by Western blot (lower) in Tca-8113 cells infected with LV-shMEK1, LV-shMEK2 and LV-CN. D, The expression levels of IRS-2 were measured by qRT-PCR (upper) and Western blot (lower) in Tca-8113 cells infected with LV-shMEK1, LV-shMEK2 and LV-CN. E, The location of ERK1/2(left) and p-ERK1/2(right) proteins in Tca-8113 cells through IHC staining. For qRT–PCR, Data represented the mean ± SEM of three independent experiments. *P<0.05

The IGF-1R is activated by IGF-I and IGF-II, resulting in phosphorylation of tyrosines in its kinase domain and the adaptor proteins insulin receptor substrate(IRS) family and Src homology and collagen domain proteins(Shc), which then transduces signals via the AKT and MAPK pathways[8, 9]. As shown in Supplementary Figure.S2, the protein expression of IGF-1R positively correlated with IRS-2 expression using Spesrman's correlation analysis (r=0.612,*P*<0.001). However, we also found that the expression of IRS-2 mRNA was significantly reduced in Tca-8113 cells transfected with si-IGF-1R compared with si-Ctrl group (Figure 6A), then we speculated that IRS-2-related transcription factors were indirectly regulated by IGF-1R-activated phosphorylation of certain protein kinases, such as ERK1/2, which could translocate to the nucleus to activates various transcription factors[26, 27]. To confirm our hypothesis that IGF-1R partly regulated IRS-2 expression through regulation of MAPK pathway, in further experiments, we found phosphorylation protein expression of MEK1/2(p-MEK1/2) and MEK1/2(p-MEK1/2) markedly decreased in Tca-8113 cells transfected with si-IGF-1R(Figure 6B). Furthermore, the packaged letiviruses of shMEK1/2(LV-shMEK1, LV-shMEK2), which specifically phosphorylate Tyr and Thr residues of the ERK1/2 proteins and the scramble lentiviral vector(LV-CN) were constructed(Figure 6C) and then infected into Tca-8113 cells, the results showed that the mRNA and protein levels of IRS-2 were significantly decreased in LV-shMEK1/2-infected Tca-8113 cells compared with LV-CN group(Figure 6D). In addition, we also confirmed that the ERK1/2 protein was mainly localized in cytoplasm, but p-ERK1/2 at both nucleus and cytoplasm using IHC staining in Tca-8113 cells(Figure 6E), suggesting that actived ERK1/2 could translocate into the nucleus and initiate transcription of genes.Taken together, these results suggested that IGF-1R regulated the expression of IRS-2 through the activation of direct IRS-2 phosphorylation and indirect ERK1/2 phosphorylation.

### IGF-1R-mediated let-7b inhibited expression of cell cycle proteins through regulation of the Akt and MAPK pathways

**Figure 7:**
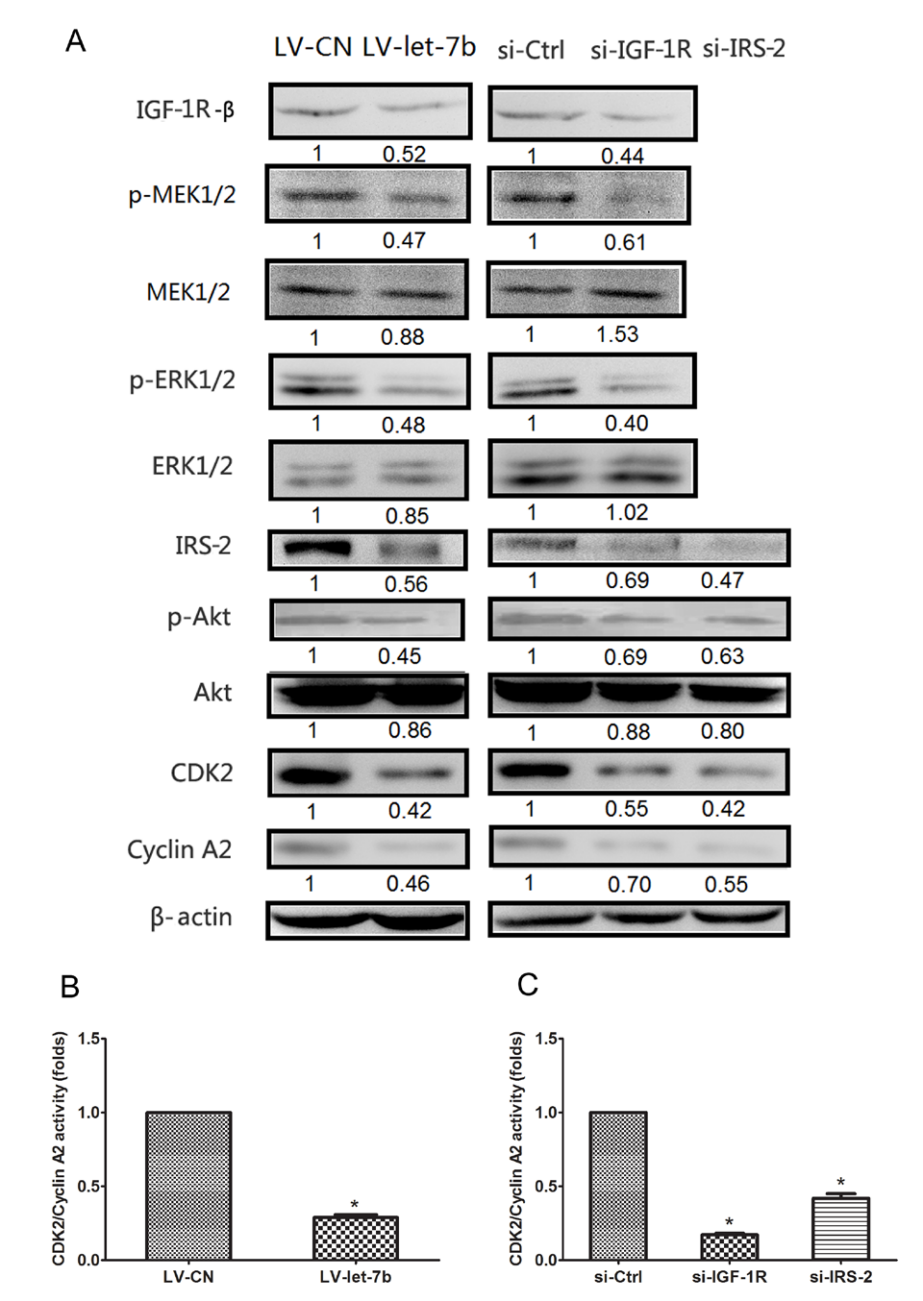
IGF-1R-mediated let-7b inhibited expression of cell cycle proteins through regulation of the Akt mediated by p-ERK1/2 A, Western blot analyses in Tca-8113 cells after infection of LV-let-7b and LV-CN and transfection of si-IGF-1R, si-IRS-2 and negative control(si-Ctrl). The amount of phosphorylated Akt (Ser-473), ERK1/2 (T201/Y204) and MEK1/2(Ser217/221) were normalized versus that of total Akt, ERK1/2 and MEK1/2. β-actin served as an internal control. B-C, Statistical results showed the normalized enzyme activities of CDK4/Cyclin A2 in cells treated with LV-let-7b, si-IGF-1R, si-IRS-2 and controls. Each data represented mean ± SEM. *P<0.05.

The Akt and MAPK pathways are involved in the regulation of cell cycle progression[28, 29] and the S-G2 phase transition of cancer cell cycle is controlled by CDK2/Cyclin A complexes[30]. To validate the downstream regulators were involved in the growth inhibition of IGF-1R-mediated let-7b, we performed Western blot analyses using relevant antibodies. The protein expression levels of IGF-1R, p-MEK1/2, p-ERK1/2, IRS-2, p-Akt, CDK2 and Cyclin A2 decreased in Tca-8113 cells infected with LV-let-7b compared with LV-CN group, however, no significant differences were observed in total MEK1/2, ERK1/2 and Akt levels(Figure 7A). Moreover, the kinase activity of CDK2/cyclin A2 was decreased in cells infected with LV-let-7b compared with control group(Figure 7B). We also found that the effects of IGF-1R or IRS-2 downregulation were consistent with those of let-7b overexpression in Tca-8113 cells transfected with si-IGF-1R or si-IRS-2 (Figure 7A and C). These findings suggested that let-7b regulated cell cycle progression through downregulation of the IGF-1R and IRS-2 mediated by activation of AKT and MAPK pathways.

## DISCUSSION

**Figure 8:**
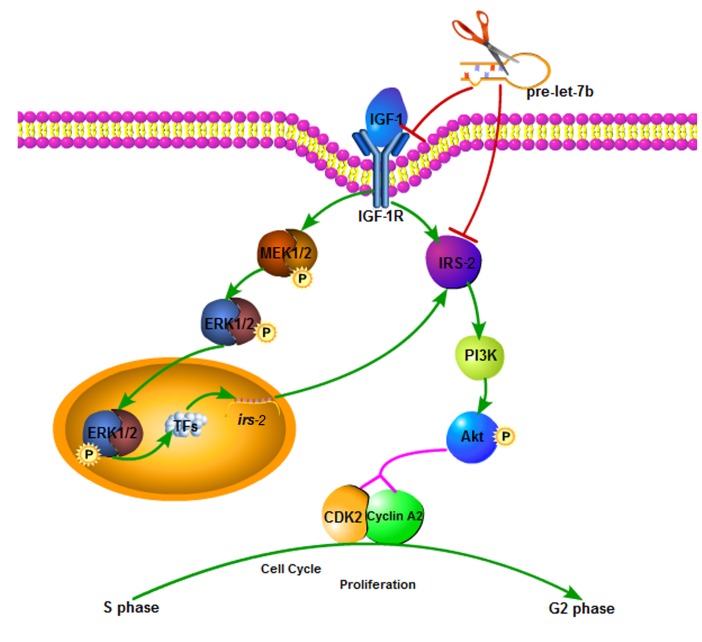
Proposed model for the effects of IGF-1R-mediated let-7b on the AKT/MAPK pathways and thereby proliferation Expression of let-7b downregulates the target genes expression, including IGF-1R and IRS-2. IGF-1R is essential factor required for the activation of direct IRS-2 phosphorylation and indirect ERE1/2 phosphorylation(p-ERK1/2) vis MAPK pathway. p-ERK1/2 is translocated into nuclears to activate the transcription of IRS-2 through regulation of transcription factors. As results of IRS-2 activation, Akt pathway involved in proliferation is promoted.

The IGF-1R is frequently overexpressed in human myeloma[13], head and neck cancer[31] and breast cancer[14], suggesting that aberrant IGF-1R expression may contribute to initiation and progression of malignancies. However, controversy remains regarding the clinical significance of IGF-1R expression, furthermore, few reports have evaluated expression of IGF-1R in OSCC patients[32]. Here, we analyzed expression of IGF-1R in a series of OSCC samples using immunohistochemistry. Surprisingly, IGF-1R expression was significantly upregulated in OSCC samples as compared with normal tissues and the association of IGF-1R overexpression with poor tumor differentiation was significant in our study, which was consistent with Kikuchi et al.'s findings[33]. But little is known about the mechanisms underlying these positive effects mediated by IGF-1R in OSCC. IGF-1R is a tyrosine kinase receptor that is activated by IGFs, the activated IGF-1R not only phosphorylates IRS-1 and SHC to activate the MAPK cascade, which stimulates cell growth and proliferation[11], but also protects from apoptosis as a result of activation of PI3K-Akt through phosphorylation and subsequent inactivation of BAD[12]. In addition, IGF-1R could promote cell motility via the phosphorylation of IRS-2[34]. Nevertheless, the effect of IGF-1R/IRS-2/Akt axis on cell growth remains uncertain. Here, we present a novel evidence that IGF-1R, for the first time, mediates crosstalk interactions between the IRS-2/Akt and MAPK pathways to promote cell proliferation, simultaneously regulated by let-7b in OSCC(Figure 8).

Although IGF-1R displays potent pro-proliferative role in different steps of oncogenesis, there still exist controversies about regulatory mechanism. Many cancer patients with type 2 diabetes has metabolic and hormonal characteristics and the hyperproliferative processes of cancer cells could be suppressed by metformin or dietary protein restriction through insulin-independent and insulin-dependent mechanisms[35,36]. Two recent studies by Fontana *et al.*[37, 38] indicated that siRNA directed against IGF-1R in the pGBMcell lines resulted in efficient reduction of IGF-1R, along with inhibition of p-Akt and induced an accumulation of cells in G1 phase in contrast to a reduction of S and G2 phases. Moreover, another *in vitro* studies[39] showed that IGF-1R inhibitor (NVP-AEW541) in BsB8 cells significantly downregulated GSK3b-mediated phosphorylation and N-Myc to induce a G1-phase arrest. In the present study, to further reveal the functions of IGF-1R in OSCC, we silenced IGF-1R by synthetic IGF-1R siRNA and observed that si-IGF-1R-treated Tca-8113 cells displayed significant reduction of IGF-1R protein level, consequently resulting in restricted cell proliferation and colony formation, blocked S/G2 transition in Tca-8113 cells. Further experiments also revealed that IGF-1R expression was positively correlated with cell cycle-related protein CDK2 and Cyclin A2 as well as the kinase activity of CDK2/Cyclin A2 complex in OSCC cells, thereby, for the first time, suggestingthat increased IGF-1R can upregulate expression of CDK2/Cyclin A2 complex via Akt pathway in the proliferative progression of OSCC.

The question of how IGF-1R becomes overexpressed remains unknown, but accumulating evidence reveals that miRNAs are major regulators of tumor development and progression. Generally, one gene can be suppressed by multiple miRNAs and one miRNA may also suppress multiple target genes[40]. For example, the expression of IGF-1R was upregulated by miR-7[6], miR-486[17] and let-7[31] in human gastric cancer, lung cancer and cervical cancer. Moreover, let-7b also acts tumor-suppressing functions by targeting the cell cycle molecules(Cyclin D1 and D3)[41], c-Myc[24] and ER-α[25]. Of note, IGF-1R was a predicted target of let-7b using bioinformatics analysis, to our knowledge, this is the first study to validate the hypothesis in OSCC. First, luciferase reporter analysis indicated that IGF-1R is indeed a specific and direct target of let-7b. Second, overexpression of let-7b in Tca-8113 cells infected by LV-let-7b decreased IGF-1R protein expression. Third, let-7b in OSCC xenografts mainly led to reducing IGF-1R protein, but not mRNA, compared with the control xenografts without let-7b through the Western blot and IHC staining, which was consistent with other previous studies, suggesting that miRNAs more often inhibit protein translation of the target mRNA, not inducing its degradation[42]. Furthermore, we also revealed an inverse correlation between let-7b and IGF-1R protein expression in clinical OSCC samples. These results indicate that IGF-1R might play a role in the development and progression of OSCC through targeted by let-7b.

Recntly, only one study provided evidence that downregulation of let-7b in oral cancer cells correlated with elevated expression levels of Dicer[23], while little is known about the functional mechanism of let-7b as a potential tumor suppressor in OSCC. Consistently, we also found that let-7b was remarkably downregulated in OSCC cell lines and clinical samples. More importantly, our data presented the first demonstration that let-7b could inhibit cell growth and colony formation, block S/G2 transition in OSCC cells and suppress the growth of xenografts, confirming the tumor-suppressive role of let-7b, as similar as IGF-1R silencing, in the progression of OSCC cancer through AKT pathway. These findings were in line with a previous studies in melanoma[41] and liver cancer[43], in which let-7b was downregulated and overexpression of let-7b inhibited cell proliferation, but the regulatory mechanisms was different. Furthermore, IGF-1R overexpression could rescue the growth-suppressive effect of let-7b. Hence, these results suggest that let-7b regulates the growth of OSCC cells mainly by targeting IGF-1R.

In addition, IRS-2, as a downstream adaptor phosphorylated on tyrosine residues by IGF-1R, transmits anti-apoptotic and pro-metastasis signals to the cell mainly through the PI3K/Akt pathway that mediates cell proliferation, migration and survival [44, 45]. Cumulative evidence suggests that IRS-2 is overexpressed in pancreatic adenocarcinoma[46], breast cancer[47] and colorectal cancer[48]. Here, we presented a novel evidence that IRS-2 expression was markedly upregulated in OSCC and that IRS-2 silencing inhibited the proliferation of OSCC cells via Akt pathway, which was parallel to si-IGF-1R. More importantly, we also found that IRS-2 was a direct target of let-7b that was identified both *in vitro* and *in vivo*. These findings suggested that IRS-2, not only phosphorylated by IGF-1R[49], but also targeted by let-7b, was involved in the regulation of cell proliferation through Akt pathway. Intriguingly, we found that IGF-1R silencing unexpectedly reduced expression of IRS-2 mRNA besides IRS-2 protein. Therefore, we speculated that IRS-2 might be indirectly regulated by IGF-1R-mediated regulators. It is well known that MAPK pathway is also considered to be a downstream signal of IGF-1R, involving in the regulation of cell proliferation, differentiation and survival[50]. ERK1/2 is specifically activated by dual-phosphorylation of MEK1/2 and serves as negative-feedback loops to PI3K by phosphorylating GAB/IRS/FRS scaffolding proteins and kinase substrates[51]. Moreover, activated ERK1/2 can translocate into the nucleus and regulate transcriptionally gene expression by phosphorylating transcription factors[27]. Based on the above studies, we, for the first time, identified that the mRNA expression of IRS-2 was downregulated via MAPK pathway in OSCC cells infected by letiviruses of shMEK1/2. Although it is uncertain which transcription factors regulate the expression of IRS-2 at the transcript level, we propose that IGF-1R regulates IRS-2 through not only direct phosphorylation, but also indirect MAPK pathway.

In summary, we, for the first time, demonstrates that overexpression of IGF-1R and IRS-2 and downregulation of let-7b are observed in OSCC cells and clinical samples. Moreover, let-7b could suppress the cell growth both *in vitro* and *in vivo* by targeting IGF-1R and IRS-2. Of note, we provide a novel evidence that IGF-1R, as a hallmark of cell carcinogenesis, could directly increase the expression of IRS-2 or indirectly upregulate IRS-2 via MAPK pathway, leading to the promotion of cell proliferation through Akt pathway, which may be a potential therapeutic strategy for the treatment of OSCC in the future.

## MATERIALS AND METHODS

### Cell lines and OSCC tissues

Tca-8113, established in Ninth People's Hospital, Shanghai Second Medical University in 1981, was purchased from KeyGEN Biotech(Nanjing, China) and grown in RPMI 1640 medium(PAA, Austria). Cal-27 cells were kindly provided by professor Erwei Song (Sun Yat-sen University, Guangzhou, China) and maintained in Dulbecco's MEM and HEK293T cells were also cultured in Dulbecco's MEM(PAA, Austria). All the media were supplemented with 10% fetal bovine serum (PAA) at 37o under 5% CO_2_.

Formaldehyde-fixed, paraffin-embedded (FFPE) tissue samples, including 64 primary OSCC and 20 normal tissues, were selected from January 1987 to June 2012 at Department of Pathology, Stomatology Hospital of Xi'an Jiaotong University College of Medicine, China. No patients received chemotherapy or radiotherapy before surgery and their clinicopathological parameters, including age, gender, histology and pTNM stage were collected. The pathological stage was defined according to the American Joint Committee on Cancer (AJCC). All the histological diagnoses for OSCC and normal tissues were confirmed by two independent pathologists. The study was approved by the Hospital Ethical Committee and informed consent was obtained from each patient prior to the study.

### RNA extraction, retrotranscription and quantitative real-time PCR(qRT-PCR)

Total RNA was isolated from the cells and frozen tissues with TRIzol reagent (Invitrogen, Carlsbad, CA,USA) and for human FFPE tissues the total RNA was isolated using the RecoverAll ™ Total Nucleic Acid Isolation Kit (Ambion, Austin, TX, USA) according to the manufacturer's protocol. All reagents for qRT–PCR were ordered from Takara (Dalian, China). For mRNA analyses, the first-strand cDNA was generated using the PrimeScript^®^ RT reagent Kit following the manufacturer's instructions, while the mature miRNA was reverse transcribed using miRNA-specific primers for quantification of let-7b. qRT-PCR was performed using SYBR Premix Ex Taq II on an FTC-3000™ System (Funglyn Biotech Inc., Toronto, Canada). The primers used were listed in Supplementary Table S1. Each measurement was performed in triplicate for each sample and a dissociation curve analysis was conducted for each PCR. U6 and β-actin were used as controls for miRNA and mRNA level, respectively. Relative quantitation was calculated using the 2^−ΔΔCt^ method.

### miRNA/siRNA transfection

Tca-8113 cells were seeded in growth medium without antibiotics, 24 hours after palting, pre-let-7b expression plasmid, constructed using synthetic oligonucleotides(Beijing AuGCT DNA-SYN Biotechnology Co.Ltd) and the pcDNA6.2-GW vector, or siRNAs against IGF-1R/IRS-2(GenePharma, Shanghai, China) was transiently transfected into the cells using Roche(No.06366236001, 04476093001, respectively) in accordance with the manufactures' protocol. pre-let-7b control(pcDNA6.2-GW vector), anti-let-7b control and scramble siRNA (GenePharma, Shanghai, China) were also transfected as negtive controls. All miRNA/siRNA transfections were performed for 24h. The sequences of small interfering RNA against IGF-1R(si-IGF-1R#1, No.2779; si-IGF-1R#2, No.2122) and IRS-2(si-IRS-2#1, No.2510; si-IRS-2#2, No.2511) were purchased from GenePharma.

### Reporter plasmids construction and luciferase assays

The complimentary sites in 3'UTR of IGF-1R and IRS-2 for let-7b were synthesized (Beijing AuGCT DNA-SYN Biotechnology Co.Ltd) and cloned into pmirGLO vector at Sac 1 and Xho 1sites(Promega), while mutated 3'UTR sequences of IGF-1R and IRS-2 were also cloned and named IGF-1R-MT and IRS-2-MT (Supplementary Table S2). The sequences of construced plasmids were confirmed by DNA sequencing(SangonBiotech, Shanghai, China).

HEK293 cells were seeded in 96-well plates(3000 cells per well) and cotransfected using 100ng of pre-let-7b expression vector along with 100ng of wild-type or mutated reporter plasmid. Twenty-four hours after transfection, firefly and *Renilla* luciferase were measured using the Dual-Luciferase Assay System(Promega), luciferase activitiy was normalized to *Renilla* luciferase activity. Each assay was repeated in triplicate.

### Lentivirus infection

The packaged letiviruses of pre-let-7b, shMEK1 and shMEK2 were constructed by GeneChem (Shanghai, China) and named LV-let-7b (LV-has-let-7b(3821-1)), LV-shMEK1 (MAPK1/GV115-RNAi-LV#1) and LV-shMEK2 (MAPK2/GV115-RNAi-LV#1), respectively. The scramble lentiviral vector LV-CN (LV-scrRNAi(PCON053)) was used as a control. For infection, the Tca-8113 cells were seeded in a 6-well plate and infected with 1 ml of viral stock containing 5μg/ml polybrene and enhanced infection solution(Eni.S) for 10 h at **37**°C, then this medium was replaced by normal culture medium.

### Cell proliferation assay

Tca-8113 cells were plated in 96-well plates at 3000 cells/well. At 24, 48 and 72h after transfection, the cell proliferation assay was analyzed using 3-4,5-Dimethylthiazol-2-yl)-2,5-diphenyl-tetrazolium bromide (MTT) assay. A 20 μl of MTT solution was added and incubated for 4h at 37°C, then the supernatant was discarded and replaced with 150μl Dimethyl sulfoxide(DMSO). Absorbance was measured at a wavelength of 492nm in a microplate spectrophotometer.

### Cell cycle analysis

At 24h post-transfection, cells were harvested and fixed with 70% ethanol at 4 °C overnight. Cells were centrifuged at 1500 rpm for 5min and incubated with 0.1mg/ml Rnase A and 0.05mg/ml propidium iodide (PI) for 30min at 4°C, then cells were analyzed by flow cytometry.

### Kinase activity assay

The activity of CDK2/CyclinA was measured using kinase activity assay kits(Genmed Scientifics, MA, USA) according to the manufacturer's instructions. Briefly, first, 500μl cell lysate were obtained from cells with transfection, then incubated on ice for 30 min and centrifuged at 13000 rpm for 5 min at 4°C. The supernatants were collected. Second, the mixture of 65μl buffer solution and 10μl substrate was incubated, 10μl reacting solution and 10μl enzymatic solution in 96-well plates at 30°C for 5 min. Finally, the optical density was immediately measured at 340 nm every 1 min for 5 min when 5μl supernatant was added into the reagent mixture. The activity was measured by calculating the difference between the absorbance value at 0 min and 5 min. Each assay was repeated in triplicate.

### Colony formation assay

The cells were seeded in 12-well plates at 2000 cells/well after 24 hours of transfection and grown for 2 weeks. Colonies were then fixed, stained with 0.1% crystal violet, counted and normalized to the control group.

### Antibodies and Western blot

Primary IGF-1R-β antibodies were from Cell Signaling Technology (#9750) (Boston, MA, USA) and Abgent(#3C8B1)(Wuxi, China), primary IRS-2 antibodies were from Cell Signaling Technology (#4502) (Boston, MA, USA) and Abgent(#EP976Y)(Wuxi, China), primary Cyclin A2 (#1547-1) and CDK2(#1134-S) antibodies were from Epitomics (Burlingame, CA, USA), primary MEK1/2(#AF6385) was from Affinity Biosciences (Cincinnati, USA), primary p-MEK1/2(#9154), ERK1/2 (#4695), Akt(#4691P) and p-Akt(#4060P) antibodies were from Cell Signaling Technology (Boston, MA, USA), primary p-ERK1/2 antibody was from Bioworld (#BS5016) (Nanjing, China) and β-actin antibody was from Santa Cruz (sc-47778).

For Western blot, the protein samples of tissue and transfected cells were harvested with RIPA buffer(Sigma-Aldrich). The equal amounts of protein lysates were separated by 7-10% SDS-PAGE and transferred to a methanol-activated PVDF membrane (Millipore, Beijing, China). The membrane was blocked with 5% non-fat milk in Tris-buffered saline Tween-20(TBST) for 2h and incubated with primary antibody overnight at 4 °C, followed by secondary anti-rabbit or anti-mouse(Pierce). Protein levels were normalized to β-actin.

### Immunohistochemistry(IHC)

The FFPE tissue samples were sectioned at 4μm thickness. Sections were deparaffinized with xylene and hydrated using graded alcohol, antigen retrieval and blocking were then performed, slides were incubated with primary antibodies(IGF-1R and IRS-2) at 4 °Covernight, followed by incubation with secondary antibodies. Detection was performed by 3,3'-diaminobenzidine(DAB) and hematoxylin. The stainning intensity was scored manually, the sample was considered as high expression if the percentage of positive cells was >50% in 5 random fields.

### Tumorigenicity assay in nude mice

Five-week-old female BALB/C nude mice were used to analyse tumorigenicity. Tca-8113 cells were infected with LV-let-7b and LV-CN and resuspended with phosphate-buffered saline (PBS), then 1×10^6^ cells were injected subcutaneously(s.c.) into the both posterior flank of nude mice. Tumor size was measured by vernier caliper every 3 days for 18 days and monitored by bioluminescent imaging using Xenogen IVIS Spectrum (USA). The mice were anaesthetized by intraperitoneal injection with 1% pentobarbital sodium (50 mg/kg). The tumor was removed after induction of deep anesthesia and the incision was closed with surgical staples. Mice were euthanized 3 weeks after the injection. Tumor volumes(V) were calculated by measuring the length(L) and width(W) of tumors and using the formula: V=(L×W^2^)/2. All animal experiments were approved by the Institutional Animal Care and Use Committee of Xi'an Jiaotong University.

### Statistical analysis

Data were presented as mean±SEM of three independent experiments at least, the Student's *t* test was used for comparisons of 2 independent groups. The relationship between the expression of let-7b, IGF-1R and IRS-2 and clinicopathologic characteristics was conducted with Chi-square test. Spearman's correlation was used to explore the association between let-7b and IGF-1R, IRS-2 expression. All tests were two-sides and differences were considered statistically significant at *P*<0.05.

## SUPPLEMENTARY FIGURES AND TABLES




