# Supplementary material for: IGF-1R, a target of let-7b, mediates crosstalk between IRS-2/Akt and MAPK pathways to promote proliferation of oral squamous cell carcinoma
Source: Oncotarget. 2014 Mar 21;5(9):2562–74. doi: 10.18632/oncotarget.1812 (PMC4058027; doi:10.18632/oncotarget.1812)
Supplement: Supplementary file 1 [file oncotarget-05-2562-s001.pdf]

IGF-1R, a target of let-7b, mediates crosstalk between IRS-2/Akt and MAPK pathways to promote proliferation of oral squamous cell carcinoma – Gao et al

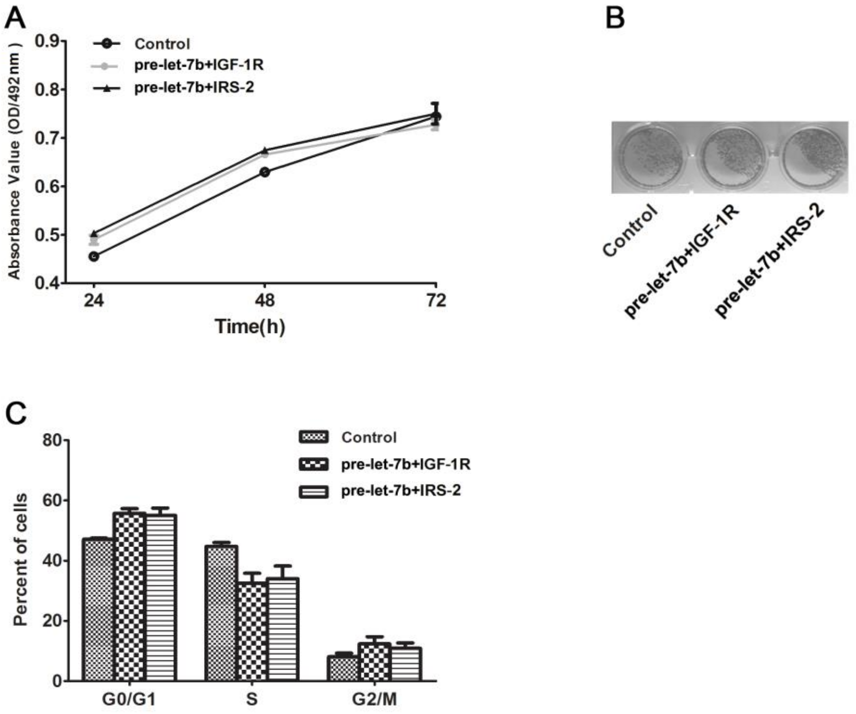

Supplementary Figure S1: IGF-1R and IRS-2 were involved in let-7b-induced growth inhibition in OSCC cells. A, At 24, 48 and 72h after transfection with let-7b, in combination with IGF-1R or IRS-2 plasmid, cell proliferation was examined by the MTT assay. All data were shown as mean ± SEM. B, Representative micrographs of crystal violet-stained cell colonies were analyzed by colony formation assay at day 15 after co-transfection. C, The histograms for cell-cycle distribution of Tca-8113 cells transfected with pre-let-7b, in combination with IGF-1R or IRS-2 plasmid, for 24h using the flow-cytometric analysis, data were presented as mean ± SEM. *P < 0.05.

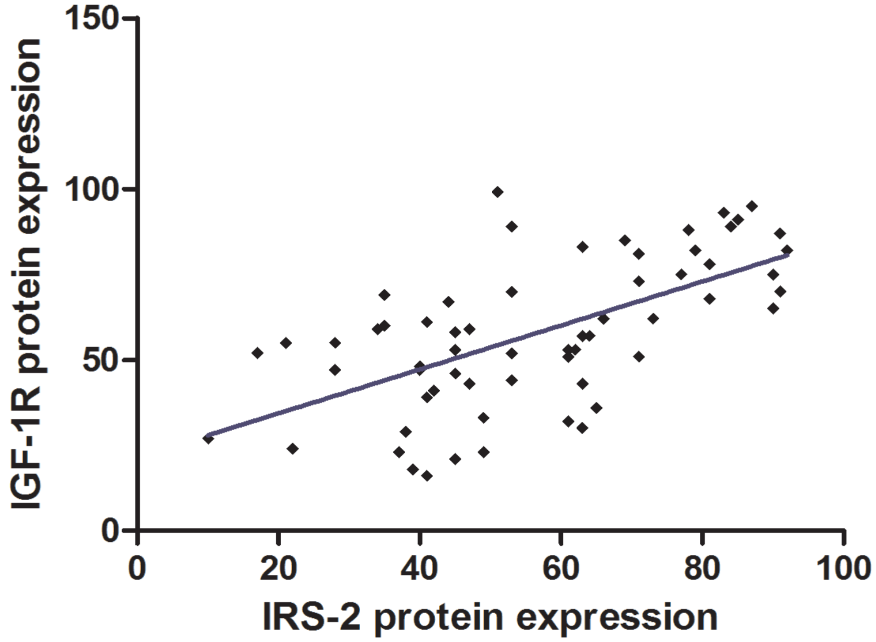

Supplementary Figure S2: There was a positive correlation between IGF-1R and IRS-2 protein expression levels. The correlation between IGF-1R and IRS-2 protein expression levels was analysed using spearman's correlation. IGF-1R and IRS-2 protein expression levels were evaluated by immunohistochemistry staining.
