# Supplementary material for: IGF-1R, a target of let-7b, mediates crosstalk between IRS-2/Akt and MAPK pathways to promote proliferation of oral squamous cell carcinoma
Source: Oncotarget. 2014 Mar 21;5(9):2562–74. doi: 10.18632/oncotarget.1812 (PMC4058027; doi:10.18632/oncotarget.1812)
Supplement: Supplementary file 2 [file oncotarget-05-2562-s002.doc]

**IGF-1R, a target of let-7b, mediates crosstalk between IRS-2/Akt and MAPK pathways to promote proliferation of oral squamous cell carcinoma – Gao et al**

Table S1. Primer sequences for qRT-PCR

| Name | Forward | | | Reverse |
| --- | --- | --- | --- | --- |
| IGF-1R | 5’-TTTCCCACAGCAGTCCACCTC-3’ | 5’-AGCATCCTAGCCTTCTCACCC -3’ | | |
| IRS-2 | 5’-TTGACTTCTTGTCCCACCACTTG -3’ | 5’-GCTGAGCGTCTTCTTTTAATGATACT -3’ | | |
| MAP2K1 | 5’-CCAAAATGCCCAAGAAGAAGCCG-3’ | | 5’-CCAAACACTTAGACGCCAGCAGC-3’ | |
| MAP2K2 | 5’-CACCATCAACCCTACCATCGCC-3’ | 5’-CCACTTCTTCCACCTCGGACC-3’ | | |
| let-7b | 5’-ATCCAGTGCGTGTCGTG-3’ | 5’-TGCTTGAGGTAGTAGGTTG-3’ | | |
| let-7b specific stem-looped RT primer: GTCGTATCCAGTGCGTGTCGTGGAGTCGGCAATTGCACTGGATACGACAACCACA | | | | |

Table S2. Sequences of recombinant plasmids

| Name | Sense Strand | Antisense Strand |
| --- | --- | --- |
| IGF-1R-WT | 5’- CCCCCCAAACATTTATCTACCTCAC -3’ | 5’-TCGAGTGAGGTAGATAAATGTTTGGGGGGAGCT-3’ |
| IGF-1R-MT | 5’- CCCCCCAAACATTTATCTAGGTCAC-3’ | 5’-TCGAGTGACCTAGATAAATGTTTGGGGGGAGCT-3’ |
| IRS-2-WT | 5’-CAAAACGACCACAGTCCTACCTCAC-3’ | 5’-TCGAGTGAGGTAGGACTGTGGTCGTTTTGAGCT-3’ |
| IRS-2-MT | 5’- CAAAACGACCACAGTCCTAGGTCAC-3’ | 5’-TCGAGTGACCTAGGACTGTGGTCGTTTTGAGCT-3’ |

Table S3. Patient characteristics and clinicopathologic correlation of let-7b expression

| Characteristics | Number of cases | let-7b expression | | P-value |
| --- | --- | --- | --- | --- |
|  |  | High | Low |  |
| Age (years) |  |  |  | 0.281 |
| ≥60 | 31 | 5 | 26 |  |
| <60 | 33 | 9 | 24 |  |
| Gender |  |  |  | 0.121 |
| Male | 43 | 7 | 36 |  |
| Female | 21 | 7 | 14 |  |
| Histology |  |  |  | 0.218 |
| Well | 37 | 11 | 26 |  |
| Moderate | 13 | 1 | 12 |  |
| poor | 14 | 2 | 12 |  |
| pT stage |  |  |  | 0.984 |
| pT1 | 16 | 3 | 13 |  |
| pT2 | 25 | 6 | 19 |  |
| pT3 | 9 | 2 | 7 |  |
| pT4 | 14 | 3 | 11 |  |
| pTNM Stage |  |  |  | 0.977 |
| Ⅰ | 15 | 3 | 12 |  |
| Ⅱ | 18 | 4 | 14 |  |
| Ⅲ | 11 | 3 | 8 |  |
| Ⅳ | 20 | 4 | 16 |  |
